# Midwifery

**Published:** 1824-09-01

**Authors:** 


### MIDWIFERY.

1. *Ascites in Pregnancy.* Mr. Langstaff (Med. Chir. Trans. v. xii.) has related an interesting case of this kind which, in a practical point of view, must be considered valuable to accoucheurs.

A lady in her ninth pregnancy, and in the 39th year of her age, appeared unusually large, and felt uncomfortable and listless, at an early period of utero-gestation. At the period of quickening, the abdominal enlargement was very remarkable. In the beginning of the seventh month, the abdominal pain and distention were so distressing as to demand local and general blood-letting, blistering, &c. The extremities became œdematous, and fluctuation was perceptible in the abdomen, but not in all parts alike, being more evident in the hypochondria, particularly the right. Calomel, digitalis, and squills were employed without effect, and the dropsical symptoms became so urgent as to threaten the life of the patient. In consultation, Dr. Farre gave it as his opinion, that the induction of premature labour was preferable to paracentesis abdominis. Dr. Davis came to the same decision. Accordingly, on the 14th March, the liquor amnii was let off. It was small in quantity. On the following day, the dropsical symptoms were more distressing than ever, and there were no signs of approaching labour. Mr. Langstaff was therefore induced to perforate the peritoneum about two inches below the umbilicus, with a moderate sized trochar. When about ten pints of transparent fluid were drawn off, the stream was checked by the uterus coming against the canula. This was obliged to be withdrawn, and an elastic gum catheter introduced in lieu, by which fifteen pints more were abstracted. Eight hours after the operation, pain was complained of over the whole abdomen, with restlessness, hot skin, quick pulse. Twenty-four ounces of blood were drawn from the arm, and was remarkably inflamed—saline aperients—five grains of calomel with the same quantity of hyoscyamus, at bed time. 21st. Bowels freely relieved—the symptoms of pyrexia and irritation the same—pulse 100, full and hard—urine high-coloured—pain in the abdomen. Twenty leeches to the abdomen, and much discharge of blood. 22d. Pain and tenderness continue, although the bowels are free, and the urine increased in quantity. Pulse 110, fuller and harder—tongue very white and dry. Thirty ounces of blood from the arm, more inflamed than ever. Saline medicines, with digitalis and 15 drops of *liq. opii sedat.* at bed time. 23d. All the symptoms ameliorated. Towards evening of this day, uterine pains came on, and the lady was delivered of a dead fœtus about four hours after the commencement of labour. The child did not appear to have advanced beyond the seventh month, and, from the appearances which it presented, must have been dead for several days. From this time every thing went on well, and no re-accumulation of water in the abdomen took place.

Mr. Langstaff, during the treatment of this case, was not able to obtain any satisfactory advice from men or books respecting the propriety of tapping. Denman decidedly sets his face against this measure. But the urgency of the case under Mr. Langstaff fairly authorized the operator, for death must have very soon taken place, had the water not been drawn off.

Professor Scarpa too, has published a memoir on this subject, and related a case successfully treated by paracentesis abdominis. The patient was 30 years of age, in her fifth pregnancy. Previously to utero-gestation she complained of constant obstuse pain in the whole circumference of the abdomen, and still more distressing pain in the loins, for which she had been bled by her surgeon to an exorbitant extent. The abdomen encreased in size with extraordinary rapidity, so that in the fifth month of utero-gestation, the patient appeared as if at the end of her pregnancy. Diuretics proved ineffectual. At the beginning of the sixth month the tumefaction of the inferior extremities, and the distention of the abdomen caused excessive dyspnœa, with frequent faintings, inability to lie down, want of appetite, loss of rest—in short, she was driven to the point of death.

In this state, Scarpa visited her, and found the abdominal integuments livid and extenuated—the umbilicus prominent—the hypogastric regions tumid and greatly elevated—the inferior extremities swollen and threatening to burst—fluctuation evident in some parts of the abdomen, though obscure in others. The imminent danger of suffocation determined Scarpa to perform paracentesis abdominis. The trocar was introduced between the edge of the rectus muscle and that of the false ribs, on the left side, when a limpid and inodorous fluid escaped, in a continued stream, to the amount of nearly 30 pints. The respiration became more free, and the patient's feelings were relieved. She fell into a sleep of three hours duration. In the course of the following night, labour pains came on—the membranes broke—and it was computed by the attendants that 15 pints of liquor amnii came away! Two fœtuses were expelled, and died in a few seconds. On the 14th day, the patient rose from bed, and resumed her domestic employments. She afterwards enjoyed excellent health.

In addition to the authority of Scarpa, Mr. Langstaff might have found another sanction for the operation he performed, in the erudite article "Grossesse," vol. 19 of the Dict. des Sciences Medicales, written by M. Marc. We shall quote a short passage from it. "*Cependant, si l'hydropisie qui complique la grossesse est assez considerable pour menacer la femme de suffocation, on ne doit pas différer de pratiquer la paracentèse.*" P. 459.

There is a passage in Scarpa's Memoir which arrested our attention, and we shall here extract it, as translated in the first volume of the Journal of Foreign Medicine, page 254.

"As for *acute* ascites, it is undoubted that the artificial and complete evacuation of the fluid contributes powerfully to re-establish the equilibrium between the exhalent and absorbent systems of the abdomen, as well as to excite the action of the secreting urinary organs. I have had frequent occasion to confirm the truth of this important point of practice in cases of *acute* ascites in children after measles, and in puerperal women, in consequence of peritonitis."

A remarkable confirmation of the above came under our notice about eighteen months ago. A man of intemperate habits and addicted to opium as well as inebriety, became affected with chronic hepatitis, as evinced by fulness and tenderness in the right hypochondrium, clay-coloured stools, scanty and lateritious urine, yellowness and sallowness of the countenance, &c. These symptoms could not be removed by the usual alteratives and common modes of treatment. There now supervened pain and tenderness over the whole abdomen, with fever, white tongue, thirst, and extreme paucity of urine. Leeches, general bleeding, aperients, diuretics, all failed, and dropsical effusion took place to a great extent, accompanied by the most distressing pains over the whole peritoneum, so that the patient could get no sleep or rest by day or by night. Under these circumstances, we directed him to be tapped in the linea alba, and about 20 pints of straw-coloured fluid were discharged. He immediately fell into a profound sleep, which lasted many hours. Diuretics and alteratives, which had no effect previously, now produced a copious discharge of urine, and from that time till this—more than twelve months—no return of dropsy has taken place. It is a curious circumstance, however, that the cellular and adipose membrane of the lower extremities, which formerly had been œdematous, is now of extreme hardness, so as to feel like so much dense and solid wood. His limbs are considerably larger in circumference than naturally, but he appears to suffer no inconvenience from this state. The integuments of the abdomen are also becoming indurated. He takes opium three times a day—about two grains each time.

P. S. We shall lay before our readers, farther on, some additional information on the subject of this paper, which reached us too late for this article.

---

2. *Cæsarean Operation successful.* M. Bosch, Surgeon to the Public Hospital of Maestricht, has recently performed this terrible operation with success. Both women were young, or in the prime of life. The pelves of both were, of course, distorted. The operations were performed in the public hospital. The incisions were in the *linea alba*, and the lives of mothers and children were preserved.—*N. Bibliotheque Med.*

---

3. *Nervous Pregnancy.* Some of our readers, not deeply versed in obstetric literature, may start at seeing the title of this short article. But many of the most celebrated accoucheurs have witnessed or written on this species of pregnancy. Baudelocque says he saw more than twenty cases of it, some of which continued for several years. M. Gerard, of Lyons, has published a memoir on this subject; but the present case, related by M. Russel of Vars, in the first number of the Gazette de Sante for the present year, is perhaps the most remarkable on record.

*Case.* Mary Gibaud, residing at Vars, department of the Charente, had enjoyed good health previous to her marriage. Shortly after this epoch, she became apparently pregnant. The menses ceased—nausea and morning vomitings occurred—the abdomen enlarged—the motions of the fœtus were felt or supposed to be felt—in short, every symptom of pregnancy was present. At the end of nine months labour pains commenced, and went on increasing for 36 hours. The midwife, unable to make out the case, called in a surgeon of great reputation. At the moment of his arrival, the patient had just fainted from a considerable uterine hæmorrhage, and the surgeon quickly proceeded to deliver. He was not a little surprised to find the uterus in an unimpregnated state. On recovering from syncope, the labour pains had gone, but in two or three hours they returned as violent as ever. The surgeon now bled her copiously from the arm, and all the unpleasant symptoms vanished. During a month she continued well—the abdomen still large. At the end of this period, the nausea and vomitings again commenced, and the usual symptoms of pregnancy went on for nine months, when the (apparent) labour pains again recurred—and again ceased after a natural and artificial loss of blood similar to that stated above. Then a month's interval—and then again symptoms of pregnancy, and so on. This state continued for three or four years, when the case getting abroad, the patient was visited by several Professors of different Universities. In the fourth year she was carried to the Hospital of Angouleme, where the physicians considered the case as dropsy, and paracentesis abdominis was performed, but without evacuating any fluid. She recovered as usual, and went on for twenty years, with all the symptoms of pregnancy, and every nine months a kind of attempt at parturition, to be relieved only by uterine hæmorrhage and a copious bleeding.

For the last five or six years the patient had every month been subject to an abscess in the left ear, from which a sanguineous pus was discharged. During the whole of the twenty years, her breasts were gorged with milk. She died in the 51st year of her age, in consequence of inflammation spreading from the ear to the brain. M. Russel and another medical gentleman examined the abdomen, where they found every organ in a state of integrity, but a considerable quantity of fat in the omentum.

Attested as the above case is, and knowing as we do, what strange vagaries Nature plays sometimes among the female race, we dare not deny the facts as here stated. But why it should be called "NERVOUS PREGNANCY" is not so clear to us. If we were called upon to give the phenomenon a name, we should apply the term *sanguineous* rather than nervous. The case is altogether curious, and may not be uninteresting to the accoucheur and the physiologist.

---

4. *Sudden Delivery.* Several cases of this kind have lately excited the attention of medico-legal writers, in this country, and of which we took notice in our last Periscope. A remarkable instance, of a similar nature, recently occured at Arras, in France. A woman, twenty-two years of age, and in the last month of pregnancy, was taken with some pain in the bowels, and thinking she was going to have a stool, repaired to the "*garde-robe.*" It was in the night. She had scarcely sat down, when her infant was born, without any pain, or the least notice, and fell into the privy below! She knew nothing of what had happened, till she heard the cries of the child. The alarm was given; but it was three hours before the infant could be liberated from its dreadful abode! It was apparently lifeless, and though, by the usual means, respiration was restored for a few minutes, yet life could not be preserved.

---

5. *The Turn of Life.* The cessation of the catamenial evacuation is always regarded with a degree of dread by the female race—especially if they happen to be affected, for some years previously, with any chronic complaint. But, even if their health be ever so good, they have a secret apprehension of the critical period—an apprehension which has, in all ages, been fostered by medical authority and observation. In these days of scrutiny and scepticism, the "turn of life" has been made the subject of enquiry, by M. de Chateauneuf, and the result is not in favour of the popular opinion—or, as it will now be called, *prejudice.* This author grounds his memoir on tables of mortality, furnished by the most respectable authorities, from which he draws the following conclusions. Between the 43d and 60th degrees of North latitude, and over a space extending from Marseilles to St. Petersburgh, the most accurate and authentic tables of records shew no other increase of mortality in females, from the age of thirty to seventy, than what necessarily results from the progress or decline of life. "*On n'aperçoit d'autre accroissement dans leur mortalité que celui necessairement voulu par les progrès de l'age.*"

At all periods within the above range, there is, in fact, according to the said tables, a greater mortality among men than women—especially between the age of forty and fifty years—hence the "turn of life," or we will say, the 45th year, is a more critical period for the lords of the creation than for the ladies. "Il resulte de ces observations que l'age de quarante à cinquante ans est veritablement plus critique pour les hommes que pour les femmes."

On these calculations and tables, we can only remark that, although they appear to prove that the general ratio of mortality among women is not increased by the turn of life, yet they do not prove that the cessation of the catamenia is unproductive of danger, and, in many instances, of death. It ought to be recollected, that the "turn of life" brings with it an immunity from some dangers, as child-bearing, for instance, and therefore, although the *general rate* of mortality may not be influenced by this epoch, the *kind of death* may vary. If, for example, as many women die, after the age of forty, in consequence of the "turn of life," as there died before that age, of child-bearing, the ratio of mortality would not be altered, and yet it would be quite certain that the critical period was a period of danger.

---

6. *Laceration of Perineum.* Mr. Churchill was in attendance on a poor woman, in the neighbourhood of Soho, in labour of her first child. After five hours had elapsed, he found the soft parts so indisposed to dilate, that he was obliged to offer resistance to the descent of the head, for two hours afterwards, fearing that the strong efforts made would rupture the perineum—"an accident that, in spite of all my endeavours to prevent, took place; for, while the woman was most uncomfortably situated, on a bed so low as to require me to kneel, to afford proper assistance, a severe pain came on, that indued her to move completely out of my reach, and before I could adjust my situation, the head of the child found its way through, being, after the usual time, followed by the body and placenta."

The perineum was torn, but the sphincter ani escaped.

We conceive that resistance, being once offered to the descent of the head, so much the greater danger is there, if this resistance be withdrawn, whether by accident or design, before complete dilatation. In the present case, it was accident, and rupture was the consequence. Mr. C. observes that—"laceration of the perineum is much more frequently the result of impatience, or of improper interference, than an accident not to be controlled." The question might here be put, would not the patient have had as good a chance, if no interference had been offered from the beginning?—We do not presume to answer this question, but only propose it to our readers.

We would advise Mr. Churchill, or any of our junior brethren, upon similar occasions to the above, to abstract blood from the arm, which we have always found to accelerate the relaxation of the parts through which the head of the fœtus has to pass.

Mr. C. very properly exhibited a powerful anodyne draught—bound the thighs together, and let the patient alone till next day. On examining the parts, he found the laceration had extended to the sphincter ani, and then branched off on each side of it, leaving a complete semicircle of that muscle unconnected with the surrounding parts. Happily the recto-vaginal septum was left entire. Mr. Churchill passed two sutures, to give the parts an opportunity of approximating and uniting. Fortunately the union was effected, and the patient is now well.

Dr. Haighton, in his lectures, drew a frightful picture of this accident, and observed, that it should never happen in the presence of a medical man. Doubtless, when the rectum and vagina are laid into one, it is a most deplorable event; but mere laceration of the fourchette, we do not consider in so very serious a light. Notwithstanding the dogma of the late Dr. Haighton, we are convinced that the fourchette may be torn in the presence of a medical man, without any neglect on his side. The total laceration into the rectum, we think, might always be prevented by proper resistance to the violent and rapid propulsion of the fœtus, on the part of the accoucheur.—*Med. Repos. No.* 6.

---

7. *Rare Case of complicated Labour.* Mr. Allan [Med. Chir. Trans. Vol. XII] has related a curious case of complicated labour, from locking of the heads of twins. The woman was thirty years of age, and this was her third labour, in which she had been eight hours, when Mr. Allen was summoned. The presenting limb was the left knee, and with it the vertex. He attempted to push up the knee, but without success. The child's ham was then hooked with the fore finger, when the knee was pulled down, and the child's head was felt to ascend. The body was delivered, but the evolution of the head was prevented in consequence of the hollow of the sacrum being occupied by the head of another child, whose body was still above the brim of the pelvis. The face of the second child was turned towards the sacrum, and its occiput closely applied to the throat of the first child. The back of the neck of the latter, was closely applied to the symphysis pubis of the mother, and its face to the back of the neck of the child whose body remained within the uterus. Under these aukward circumstances, it was found impossible to push up the head next the sacrum, without carrying the other before it—and every attempt to extract that which was next to the pubes had the effect of pressing the other so forcibly downwards, as to threaten a rupture of the perineum. Mr. Allan was a little embarrassed, but soon made up his mind as to what he should do. Before he could put his intention into execution, however, a violent parturient effort expelled both heads at the same time. Both children were dead. The mother had a smart attack of hysteritis, requiring the abstraction of seventy-six ounces of blood, and other means for its reduction.

The mode of proceeding which Mr. Allan intended to pursue, had nature not expelled the twins, was to detach the body extruded, from the head within; and then push up the latter out of the way, delivering the child whose head presented by the forceps. The detached head must afterwards have been delivered with the forceps, or in whatever way the operator found most convenient. As an addition to this plan, we would suggest that, before pushing up the detached head, a piece of garter or tape should be passed through the lower jaw, or, in some manner, be secured to the trunkless head, in order to extract it afterwards, and thus save the second introduction of the forceps, or other instrument.

8. *Spontaneous Rupture of the Uterus, in the Fourth Month of Utero-gestation.* There are few, perhaps no well-authenticated instances on record, of *spontaneous* rupture of the uterus at so early a period as that above-mentioned. The following case, however, is authenticated beyond all possible doubt. It was read at the " Societe de Medecine de Paris," by Dr. Duparque, and since published in several Journals. We take our account from the Gazette de Sante of March last.

A married woman, thirty-three years of age, had enjoyed good health, and borne several children. In her second pregnancy, she experienced a fall, which produced abortion, and, from that period, she was affected with menorrhagia—except when pregnant, at which periods, the menorrhagia disappeared.

In the fourth month of her fifth pregnancy, being in good health, and rather embonpoint, she gave way to a most violent paroxysm of rage (to which, indeed, she was very prone) on the subsidence of which, she complained of an acute pain in the abdomen, with a feeling as if something had snapped. She passed the night, however, in tranquillity, and next day attended to her domestic concerns. The second night was also passed quietly, but, on the third morning, she perceived some spots of blood on her linen. She walked a considerable distance this day, to consult a midwife, who advised her to go home, and keep quiet. On returning home, at half-past twelve, she ate with a good appetite. At four, p. m. while using some exertion, she was suddenly seized with violent pains in the abdomen, which continued, and were exasperated when she took any thing to drink. The stomach rejected every thing that was swallowed. She had a little rest in the course of this night. On the fourth morning, at six o'clock, she suddenly turned pale, became oppressed, and after hiccuping twice or thrice, expired, perfectly sensible to the last moment.

*Dissection, Seven Hours after Death.* The abdomen was tense, but not sonorous. When opened, there flowed out a quantity of black and partially coagulated blood, with which the lower half of the abdominal cavity was filled—being about four pounds in all. In the abdominal cavity was also found a male fœtus, of about four months. Following the umbilical cord, it was traced into a rent in the superior and posterior part of the uterus, of about two inches in extent, around which rent the parietes of the uterus were thinner than in any other portion of that organ, and there were here a congeries of dilated vessels that gave the part a dark colour, as compared with the surrounding structure. A portion of placenta, the size of a pullet's egg, projected through the rent, the remainder of it being within the uterus, adherent to its internal surface, except at the part where the parietes of the organ were thin, as above-mentioned. There was no other morbid appearance—no trace of peritoneal inflammation.

Dr. Miquel, editor of the Gazette, and who is an accoucheur himself, has hazarded some reflections on the above case. He thinks, and probably with justice, that the thinning of the uterine parietes, and the varicose state of the vessels around the rent, shewed lesion of old standing—probably to be traced to the fall, which produced abortion, some years previously. Was it to this cause, he asks, that we are to attribute the habitual menorrhagia, to which she was subject, except during the periods of utero-gestation? To the next conjecture which Dr. M. hazards, we cannot give our entire assent—namely, that the rupture of the uterus took place during the paroxysm of rage, on the first day of the narrative, but that the rent was plugged up by a portion of fœtus or placenta, for a space of thirty-six hours, thus preventing hæmorrhage, and permitting the woman to follow her domestic occupations, without inconvenience. To us, this does not appear probable. We think there would have been a *continuance* of pain during life, and that there would have been traces of peritoneal inflammation after a solution of continuity of thirty-six hours duration, discoverable on dissection. We are of opinion, that the rupture of the uterus took place at four in the afternoon, fourteen hours before her death. We find that, at that hour, she was suddenly seized with violent pain, which continued, accompanied by other alarming symptoms, and soon terminated in death.

---

9. *Melange de Chirurgie Etrangere, par une Societé de Chirurgiens de Geneve.* Vol I. pp. 476. Geneve, 1824.

ART. I. *Sur la Grossesse accompagnée d'Ascite.* Par le Chevalier ANTOINE SCARPA, &c. A society of surgeons at Geneva, among whom are the two Maunoirs, Mayor, Peschier, Morin, Dupin, and Olivet, have undertaken to collect, and translate into the French language, the most interesting surgical memoirs, published in the various countries and tongues surrounding Switzerland. The first volume of this enterprize is now before us. A considerable portion of it is occupied with Memoirs which have appeared in this country, and more especially in the Medico-chirurgical Transactions of London. Of these it will be our hint to speak, in this place, as we give ample analyses of them, as they appear. Of the foreign papers, of any interest, contained in the Geneva Melange, we shall endeavour to lay a full account before our readers, beginning with the venerable chirurgical patriarch of Pavia, whose memoir stands first on the list, in the volume before us.

### PREGNANCY COMPLICATED WITH ASCITES.

Those who have read Mr. Langstaff's interesting case of this kind, published in the last volume of the Medico-chirurgical Transactions, must feel with him, how desirable it is to have as much information as possible collected on this obscure point of obstetric surgery. An account of Scarpa's memoir is contained in the first volume of the quarterly Journal of Foreign Medicine; but, in the present memoir, there are cases and observations added, that are of more importance than the original—and it is on these we shall principally dwell.

A collection of water in the uterus, or in the cavity of the abdomen, during pregnancy, becomes a very serious accident, in consequence of the swelling of the lower extremities, distention of the abdomen, displacement backwards of the abdominal viscera, embarrassment of the diaphragm, constriction in the thorax, and consequently oppression of the organs of respiration. When the water is in the cavity of the uterus, the case is not near so formidable as when the collection is in the cavity of the peritoneum.

*Case* 1. This case, related by Scarpa in his memoir, and translated into the first volume of the Journal of Foreign Medicine, will be found at page 492 of our present Number, along with Mr. Langstaff's case. We shall therefore only refer to it and pass on to the new matter now brought forward.

*Case* 2. By Dr. Cruch, Hospital Surgeon of Pavia. Dr. C. observes, that the success attending Scarpa's operation is hardly sufficient to authorize us to puncture the abdomen in the place pointed out by that venerable surgeon, until we have more facts collected on this point. The following case is therefore deserving of attention.

Mary Gregnani, ætat. 29, was received into the Hospital of Pavia, on the 30th April 1820, being then about five months gone with child, and evidently labouring, at the same time, under ascites. She had had a protracted intermittent fever previously, which gave way to the use of the bark, soon after which the ascites appeared, and increased so much as to greatly incommode her breathing. She could now rest better lying on her face than in any other position. Diuretics, drastric purges, and venesection were tried, but without affording any relief. By the latter end of August the accumulation of water threatened suffocation, and as the patient appeared to be in a condition similar to that of Scarpa's, our author determined on paracentesis abdominis, in the left hypochondriac region, as Scarpa had directed. The operation was performed on the 7th September, in presence of M. Panizze, Professor of Anatomy. Twenty-five pints of water were drawn off, having a slight greenish tinge. Great relief was immediately felt by the patient. Although the quantity of water left in the abdomen was very trifling, yet it very quickly re-accumulated, and in 24 hours the abdomen was again considerably distended. In the night of the 9th the wound opened, and a good deal of water escaped spontaneously. In the night of the 10th labour came on, and Gregnani was delivered of a living child. The labour pains then ceased, and the placenta was not expelled. Flooding succeeded, and went on to a great extent before any attempt was made to extract the after-birth. When the attempt was made, it failed, in consequence of a partial adhesion of the placenta to the uterus, which the surgeon was afraid to separate, lest he should injure the uterus! The poor woman died of internal hæmorrhage in the mean time.

*Dissection.* Eight pints of water in the peritoneal cavity—no ap-appearance of phlogosis in the peritoneum or other part of the abdomen. The uterus was as large as in the sixth month of pregnancy. On its internal surface the placenta adhered for the space of about three inches. In attempting to separate this adhesion, it is said that lacerations of the internal parietes of the uterus took place.

On this case we beg to offer a very few remarks. We think the operation was performed too late—not that the patient's death was occasioned by it, but an earlier evacuation of the water would have prevented a great deal of suffering. In the second place, we cannot but condemn the inertness of the practice, in suffering a woman to die of uterine hæmorrhage, while the placenta remained in the womb. As to the lacerations which took place (or are said to have taken place) during *post mortem* separation of the placenta, we regard them as no argument against the propriety of separation *during life.* Many parts of the body will give way to a slight force, after vitality has ceased, which would have manifested great power previous to that event. In short, the woman could only have died, whatever happened during the extraction, and without this process there was little chance of arresting the hæmorrhage.

M. Peschier, who is the translator of the memoir, remarks, that he has very frequently checked uterine hæmorrhage, by exhibiting the extract of rhatany, in doses of one drachm every hour. We are not aware that this medicine has ever been exhibited in such cases in this country.

*Case* 3. (By the same author.) M. L. Mandilini, 31 years of age, of plethoric habit, had, like the preceding patient, been long harrassed with intermittent fever, accompanied by distressing irritability of the stomach, and followed ultimately by icterus and enlargement of the liver. From these, however, she was recovered pretty well. She became pregnant, and was delivered of a dead child. In October, 1819, she was attacked with discharge of blood from the uterus, which was repressed by cold applications and astringents. In November of the same year she was threatened with hæmoptysis, which was averted by bleeding, digitalis, and nitre. In June 1820, Mandilini became pregnant a second time, the utero-gestation going on regularly till the sixth month, except that the patient could not lie on the left side. Towards the end of October she complained of frequent pains in the lower belly with desire to make water, cough, and difficulty of breathing. The abdomen was now observed to be much larger than in ordinary states of pregnancy, six months advanced, and, in fact, ascites was clearly ascertained to exist, though to no great extent. *2d November*. The abdomen was now excessively distended and almost livid—the breathing very difficult—the lower extremities greatly swelled. Paracentesis (in the same place as before) was performed, and 30 pints of limpid and inodorous water were drawn off, with great relief to the patient. Still the uterus appeared much larger than natural at such a period of pregnancy. Two hours after the operation, slight uterine pains came on, during the continuance of which, about six pints of watery fluid came away *per vaginam.* After this, blood was discharged, partly coagulated, partly fluid, followed by much faintness, from which, however, she soon recovered. On the 9th November real labour pains set in—the membranes presented themselves in a bag—and in four hours two still-born children were expelled, in size corresponding with six months utero-gestation. The after-birth followed, without any assistance. From this time the patient recovered well, and had no return of the dropsy.

It will have been observed that in all the three cases related, parturition very quickly followed the operation.

*Case* 4. (By Charles Maunoir.) Madam V———, pregnant four months and a half, applied to our author on the 11th January, 1819, complaining of acute pains in her stomach—inexpressible anguish in the chest, augmented by the slightest mental agitation—tightness in breathing. The pulse was small—the abdomen larger than it ought to be at that period of pregnancy—face pale—eye-lids swelled. Diuretics—fomentations to the abdomen. 18th January. Swellings about the spine, chest, and parts of generation—dyspnœa very troublesome—pulse small and hard—urine scanty and high-coloured. Venesection. The breathing became freer after bleeding—yet the swelling gained ground—especially about the groins and labia pudendi. Scarifications gave vent to much discharge, and produced great relief. 27th. When visited, the patient expressed great anxiety, but no actual pain to announce an approaching labour. In the course of three hours, however, labour pains set in, and the accouchment was speedily over. The fœtus was in a state of putrefaction—the placenta followed. There was no discharge, either of blood or water. The abdomen was still dropsical, and the fluctuation evident. Diuretics and purgatives were administered. On the 3d day, a diarrhœa supervened—and the urine began to flow in abundance. The anasarca diminished. In twelve days after the accouchment, no fluctuation was perceptible in the abdomen. Afterwards tonics were administered, and the patient got quite well.

*Case* 5. (By the same author.) It is rare, he observes, to find pregnancy take place in a person who has frequently undergone and is still undergoing paracentesis abdominis. Here follows a case of that kind.

On the 20th April, 1804, our author was called to perform the operation of paracentesis on a woman residing in the country. She informed him she was pregnant, as she had not menstruated for some time. On examination, however, Mr. Maunoir came to the conclusion that she was *not* pregnant. Fluctuation was very evident in the abdomen ; and engorgement of the liver was suspected. Paracentesis was performed on the left side, and 25 pints of water were drawn off, limpid but viscous. Diuretics were ordered, and the woman was soon about her domestic concerns. The abdominal effusion, however, returned, accompanied by inclination to vomit—frequent desire to urine, but the discharge scanty, in proportion to what she drank. In two months the abdomen was so enlarged that it was necessary to tap her again, when nearly the same quantity was drawn off. She still insisted that she was pregnant, but examination belied the idea. In a month, the operation again performed. At this time our author lost sight of the patient, and did not see her till the beginning of January 1805. A surgeon in the neighbourhood had attempted to draw off the water from the abdomen several times in the interval, but had only succeeded once. At this period the woman was found to be actually pregnant, having become so about the middle or end of October 1804. She was now much emaciated, but had no vomitings. Paracentesis for the 5th time (not counting the unsuccessful attempts) and milk diet recommended, with diuretics of digitalis and cream of tartar. But the accumulation of water was very quick, and she was obliged to be tapped, often at intervals of three weeks. On the 27th June, after a quantity of water had been drawn off, a tumour above the pubis was very perceptible. On the 15th July, she was delivered of a living child, the abdomen still preserving a great size. A diarrhœa, which could not be restrained, now came on, and reduced the patient to the lowest ebb. At this time, an erysipelatous inflammation appeared on the parietes of the abdomen, and threatened gangrene, but death put an end to her sufferings eight days after parturition. On dissection there was ovarian dropsy as well as ascites and anasarca.

